# 644 Burn Violence Against Women: The Tip of the Iceberg from a Country’s Nation-wide Burn Centers

**DOI:** 10.1093/jbcr/iraf019.273

**Published:** 2025-04-01

**Authors:** Yvonne Singer, Lincoln Tracy, Claudia Malic, Belinda Gabbe, Lisa Martin, Heather Douglas

**Affiliations:** Griffith University; Monash University; Ottawa Children’s Hospital of Eastern Ottawa; Monash University; University of Western Australia; University of Melbourne

## Abstract

**Introduction:**

Violence against women is pervasive. An estimated one in four women aged over 15 years old in this country have experienced physical or sexual violence by an intimate partner. Recent high-profile cases of homicidal burn violence perpetrated against women have shocked the nation. Since 2012, the Counting Dead Women project has tallied 30 women whose deaths involved fire-related violence. Currently however, there is a paucity of evidence about the frequency and burden of burn violence against women in this population. The aim of this study was to describe and compare the frequency, sociodemographic characteristics, injuries, and outcomes of women admitted to nation-wide burn centres with burns caused from suspected violence with women with unintentional burns.

**Methods:**

Data were extracted from the country’s bi-national burn registry for women (≥ 18 years) admitted to nation-wide burn centres, between 2009 and 2022, with burns caused by suspected violence or unintentional burns. Statistical analyses were not performed due to substantial differences in comparator group sizes that would have greatly reduced the statistical power, reliability, and validity of results. Socio-demographic profiles, injury characteristics, and in-hospital outcomes were described and compared.

**Results:**

Between 2009 and 2022, 157 women (2.5%) were admitted with burn injuries from suspected violence, and 6105 women (97.5%) were admitted with unintentional injuries. Women with burns from suspected violence were younger (median 36 vs 43 years) than women with unintentional burns, greater proportions sustained fire-related burns (38.1% vs 25.3%) with gasoline fuelling the flames (80.0% vs 38.0%), their injuries were more severe (median 2.0 vs 5.0 %TBSA), more likely to involve their head (42.3% vs 16.4%), and a greater proportion of them died (4.5% versus 1.5%). The perpetrators of burn violence were most often women’s current or ex-partners.

**Conclusions:**

Our study contributes to an emerging and disturbing body of international literature demonstrating the confronting nature and consequences of burn violence perpetrated against women. Our findings are likely an underestimation of the true number of women with burns caused from violence admitted to burn centres, as women do not always disclose violence.

**Applicability of Research to Practice:**

Whilst the wider problem of ending violence against women currently appears intractable, there is a valuable opportunity available now for burn centers to better protect women, by building capacity and skills within burn teams to be able to better identify women at risk and respond to women who disclose violence.

**Funding for the Study:**

NIL

